# A Cooperative Optimization Model for Variable Approach Lanes at Signaled Intersections Based on Real-Time Flow

**DOI:** 10.3390/s24175701

**Published:** 2024-09-02

**Authors:** Zhiqiang Zhu, Mingyue Zhu, Miaomiao Liu, Pengrui Li, Renjing Tang, Xuechi Zhang

**Affiliations:** 1 Platform of Transport Technology Thinktank, Research Institute of Highway, Ministry of Transport, Beijing 100088, China; zzq11211@163.com (Z.Z.); zq.zhu@rioh.cn (X.Z.); 2 School of Transportation Science and Engineering, Beihang University, Beijing 100191, China; zmymoon@buaa.edu.cn (M.Z.); 19374145@buaa.edu.cn (P.L.); renjingtang@buaa.edu.cn (R.T.)

**Keywords:** variable approach lane, signal control, cooperative optimization model, average delay difference model, threshold conditions

## Abstract

To resolve the congestion caused by imbalanced traffic at intersections, this paper establishes a model of the average delay deviation with the minimization of the average delay in the approach as the optimization objective. Then, the signal control scheme is further optimized based on the variable approach lanes setting. First, we investigate the threshold conditions for setting the VALs under different flows in a single approach direction. The results show that when the ratio of left-turn traffic exceeds the threshold range of 0.20~0.28, the function of the VALs needs to be changed from straight to left-turn. Then, based on the improved Webster’s formula, an optimal timing method that aims at minimizing the average vehicle delay, minimizing the queue length, and maximizing the capacity, is proposed. Finally, taking the actual Huangke intersection in the Hefei demonstration area as an example, three schemes are compared and analyzed in the case of a VAL at the intersection. The results show that under the cooperative optimization scheme proposed in this paper, the travel time and the efficiency of the intersection could be reduced by 18.7% and 9.9%, respectively, when compared with the original and Webster’s schemes.

## 1. Introduction

Urban road intersections are accident-prone traffic areas due to their high traffic flow and complex traffic characteristics. All directions of traffic and pedestrians flow in this interweaving mix, resulting in low efficiency and traffic congestion becoming increasingly prominent. Urban road intersections have different directions of traffic flow fluctuations and an unbalanced distribution in space and time, so the left turn and straight lane cannot undergo full and balanced use. Variable approach lanes (VALs) are considered one of the managed lane facilities to mitigate traffic congestion on a roadway. VALs at intersections can adjust the turning function of each guide lane in the approach, mainly to address the uneven distribution of traffic in different directions at the intersection. They can be used to make full and reasonable use of the limited intersection space–time resources under the existing road supply conditions and improve the traffic efficiency of urban road intersections. As the first city in China to successfully apply variable lanes, Jinan has achieved remarkable results in relieving over-saturated congestion at left-turn intersections on the city road network during morning and evening peak hours and improving the efficiency of the entire city road network.

VALs are cost-effective for expanding road capacity without constructing new infrastructure. The literature on VALs has grown over the last two decades. Harvey et al. [1] studied the signage marking system for dynamic division of lane functions with an example of an actual intersection in Houston, TX, USA, but did not specify the control strategy for VALs. Hoose, H.J. [2] formulated the control strategy for VALs according to the traffic management rules in 2005, which can effectively guarantee the normal passage of road users and ensure their safety. Wolshon, B. and Laurence Lambert [3] analyzed the conditions of use of VAL technology and discussed its traffic facilities. To better utilize the efficiency of signal intersections, the cooperative model between VAL design and signal control is mainly proposed [4].

However, there is insufficient research on the combination of real-time flow and signal control, as well as quantitative analysis of variable lane thresholds. Further research is needed to explore the cooperative optimization model for VAL and signal timing at intersections based on real-time flow. Therefore, based on the above research, this paper uses real-time traffic flow to determine the moment when the variable lane function needs to be changed and provides a generic flow range for the function shift. Webster’s delay formula is modified and integrated into the multi-objective optimization model of this paper. The optimal timing scheme of multi-factor hybrid linear programming is proposed synchronously based on the function change in VALs in real-time flow, and its feasibility and implementation effects are studied through simulation and examples to ensure the improvement of intersection traffic efficiency.

The key contributions of this study are outlined as follows:

1. A functional shift setting of the VALs in signalized intersections is constructed. Based on the real-time traffic flow at the intersection, a corresponding VAL threshold recognition scheme can be generated in real time, and corresponding flow discrimination can be performed to transform the VAL function.
2. Cooperative control optimization of the signal control scheme based on setting VALs is proposed to maximize the intersection traffic benefits. A multi-objective optimization model of intersection VAL signal timing with the objectives of minimizing the average vehicle delay, minimizing the queue length, and maximizing the capacity is established.

The outline of this paper is as follows: Section 2 presents the related works. Section 3 first describes the research scenarios and basic assumptions. Section 4 explains how to select the model. Section 5 introduces the model establishment. Section 6 describes the experimental setting and presents the simulation results. We conclude this paper in Section 7.

## 2. Related Works

Relevant prior work includes studies of switching the functions of VALs and cooperative optimization of spatial and temporal resources at intersections.

### 2.1. Switching the Function of VALs

Gong et al. [5] studied the algorithm for switching the direction of passage of VALs in tidal traffic. Assi et al. [6] proposed a variable lane function conversion scheme based on real-time traffic data, with the left-turn traffic flow at the intersection approach as the variable and the minimum total delay of vehicles at the intersection as the optimization objective. This finding represents a plausible, quick method to instantly predict the optimum lane group in the field using the percentage of turning movements at the approach without conducting massive calculations. Dante et al. [7] used cellular automata and real traffic data to model adaptive reversible lanes. The model shows that adaptive reversible lanes can improve traffic flow by up to 40% compared to conventional reversible lanes. Jiao et al. [8] used saturation degree as the direction-change switch condition of reversible lanes. If the saturation degree exceeds 0.9, turn on the direction-change switch. A dynamic prediction model of intersection traffic flow was constructed to predict all directions of car traffic in the next few cycles to determine whether the saturation threshold condition is met.

Some researchers also proposed corresponding traffic management methods for VALs to increase the road capacity and optimize the road network configuration. Afandizadeh et al. [9] developed a road network optimization model, and the upper-level problem is to minimize the total travel time of network users, while the lower level is a traffic assignment model. Nassiri et al. [10] studied the real-time adjustment technology of VALs. The offline scheme is used to adjust the VALs through a logit model, and then, the lane direction is adjusted in real time. Xie et al. [11] built an evacuation network optimization model based on VALs by integrating the strategy of eliminating conflicts between VALs and intersections. Based on the developments in automobile technology, Hausknecht et al. [12] proposed the use of traffic sensors to record traffic conditions in real time and then transmit the data to the traffic management department, which changes the direction of the road lanes in real time according to changes in traffic flow. The setting conditions of VALs are determined by analyzing the characteristics of traffic volume over one day [13]. Liu et al. [14] introduced a Predictive Empowered Assignment scheme for Reversible Lane (PEARL). Malekzadeh et al. [15] presented optimal internal boundary control for lane-free automated vehicle traffic. Jin et al. [16] studied a novel traffic control concept of internal boundary control for bi-directional lane-free traffic of automated vehicles on freeways. Malekzadeh et al. [17] presented a microscopic implementation of internal boundary control in lane-free environments. Xie et al. [18] constructed a reversible lane control model based on short-term traffic flow prediction.

### 2.2. Cooperative Optimization of Spatial and Temporal Resources

In previous studies, the VALs were found be effective in enhancing traffic efficiency and capacity. However, in some practical scenarios, optimization in conjunction with signal timing can effectively improve temporal efficiency. In previous research on the signal timing optimization of VALs at intersections, many researchers only took the minimum delay or the maximum traffic volume as the optimization goal in traditional studies and used the Webster signal timing method to judge the change in variable lane function under the condition of unsaturated traffic flow. Many existing studies have proposed different vehicle delay optimization models, which normally aim at the minimization of queue length or maximum traffic capacity at intersections, effective use of lane space, and service level as the optimization objective. At the same time, many researchers, such as Zhou et al. [19] and Liu et al. [20], have carried out multi-objective signal timing optimization, and traffic flow efficiency has also been greatly improved compared with the original signal timing. However, in the multi-objective optimization model, some factors are not considered comprehensively and are not suitable for complex intersections.

He et al. [21] established a multi-period lane switching and signal phase combination optimization model. Optimization and simulation results indicate that the new method can obtain better operation performance than the lane-based method. Cui et al. [22] investigated the problem of optimally allocating input rates to entry links and simultaneously finding a stabilizing signal control policy with phase fairness. Lu et al. [23] proposed a dynamic reversible lane assignment method for approaches that consider the game equilibrium between road users and traffic controllers. Meanwhile, a Bi-Level Programming Model is used for allocation optimization to minimize the total queue length of signalized intersection approaches. Hong et al. [24] developed a Bi-Level Programming Model to ensure minimum construction costs and a minimum total travel time on the road network. Three heuristic algorithms were used to solve the problem. The results show that the reasonable addition of temporary reversible lanes can reduce the total system travel cost, solve the temporary tidal traffic phenomenon, and alleviate traffic congestion. Wong et al. [25] proposed an optimization model based on lane turn directions, aiming at maximum traffic capacity, minimum cycle length, and minimum delay at intersections, using heuristic algorithms and case analysis to verify the feasibility of the model. Zhuo et al. [26] investigated the effect of CAV platoon configurations at a typical isolated roundabout in a mixed traffic environment to enhance efficiency. Xuan et al. [27] designed a pre-signal system and set a reasonable length of the waiting area allowing the traffic capacity of the intersection to be significantly increased. Fu et al. [28] used the neural network control method to carry out the adaptive signal control method of lane change under dynamic flow based on actual traffic data. Zhao et al. [29] demonstrated the application of microsimulation software at pre-signal-controlled intersections. On the hardware front, real-time information on AV position and speed can be obtained through connected technologies and multi-sensor integration methods [30]. Xue et al. [31] designed an observer-based event-triggered adaptive platooning control algorithm for autonomous vehicles (AVs) with motion uncertainties (e.g., unknown AV mass, internal resistance, and external disturbances). Tian et al. [32] proposed a traffic organization method for setting VALs at intersection exits. Zhao et al. [33] proposed a special left-turn exit lane design algorithm for the large left-turn traffic flow. The intersections with the left-turn exit lane were redesigned, and the signal control timing scheme was optimized. Qu et al. [34] analyzed the main traffic flow according to the unbalanced coefficient. A half-cycle green wave co-optimization strategy is proposed based on the characteristics of major traffic flows. Zhao et al. [4] proposed a bi-level model-interactive relationship between VAL design and signal control and carried out a case study for a road in Wuhan, China. The design plan for the goal, combined with the design plan of the VALs, optimizes the signal timing plan of the intersection. A real-life case is modeled in VISSIM to simulate the design scheme of VALs and verify the reliability and effectiveness of the bi-level model. The results showed that the average vehicle delay at the intersection was reduced by 20.65% after the bi-level model was optimized. Chen et al. [35] proposed a model of the intersection capacity, delay, and vehicle emissions of the VALs. The results show that the intersection capacity is increased by 14.57%, and the average vehicle delay and vehicle emissions are reduced by 21.13% and 13.43%, respectively.

## 3. Model Assumption

### 3.1. Research Scenario

**Figure 1:**
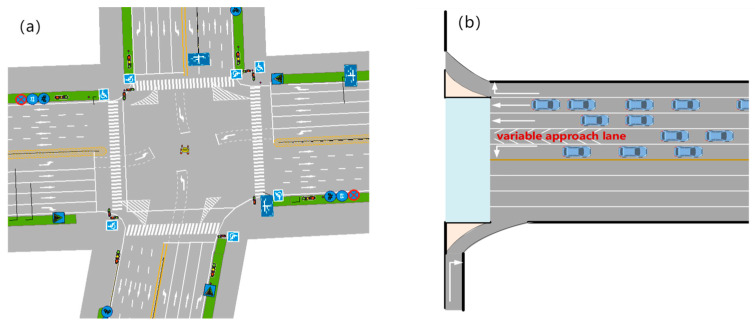
Intersection layout. (**a**) Overall layout. (**b**) Channelization design.

This paper chooses the Huangke intersection in the Hefei Demonstration Area as the research object. The intersection has a large traffic flow, and at the same time, the shape of the intersection is relatively regular, which is suitable for the setting of VALs. The intersection’s layout is shown in Figure 1. Variable guidance lane technology can change the direction of the vehicle in real-time according to the traffic turning demand; that is, the lane has a non-fixed vehicle movement or turning function. Variable guideway lanes are flexible and differ from conventional lanes in that they contain several diagonal lines, commonly referred to as “sawtooth” markings. The intersection has five lanes in the east approach direction, with motor and non-motor vehicle separation. The channelization is designed from inside out with left-turn lanes, variable guide lanes, straight lanes, and right-turn lanes, in that order. Right-turn vehicles are not considered, and the initial functional attributes of the variable approach lane are straight. The lane settings at the intersections are all set up according to Figure 1a, which shows the real existing site conditions. The data are available on the OpenITS website (http://www.openits.cn/openData2/710.jhtml, accessed on 19 November 2021).

The intersection signal timing scheme is shown in Table 1, with a cycle length of 106 s.

### 3.2. Research Hypothesis

Variable approach lanes can compensate for the shortcomings of insufficient utilization of left-turn or straight lanes caused by fixed-turn lanes at intersections through the adjustment of lane function conversion. To ensure the safe and efficient passage of the vehicles, when the left-turn and straight traffic flow reach a certain threshold, the functional attributes of the variable approach lane should be changed to achieve optimal matching of lane function and traffic flow.

The threshold curve for the change in the functional attributes of the intersection variable approach lanes is determined using the approach average delay value as the discriminant and the approach straight and left-turn traffic flow as inputs. Because right-turn traffic is not controlled by signals, vehicle delays in the right-turn lane can be ignored, and only average vehicle delays are calculated for the left-turn lane group and the straight lane group.

The installation of variable approach lanes requires specific road traffic conditions, including the design of the approach channelization, the signal timing scheme, and the flow direction of each approach lane. This paper takes a cross-signal intersection as the object of study, with the following basic assumptions:

(1) The intersection traffic flow has time and directional imbalance characteristics, which need to be solved with the application of variable guidance lanes.
(2) There is at least one left-turn lane and one straight lane at the intersection approach, and the number of lanes with variable approach lanes in the approach is at least 3, which is a typical application scenario for setting variable guidance lanes.
(3) Intersection signal timing is set to the standard four phases, with left turn and straight phases. Right-turn vehicles are not controlled by signal constraints under Chinese traffic law; therefore, right-turn vehicles are not considered in this paper.

These assumptions and constraints are the basic requirements for setting up VALs, and the signals are set up according to the current common forms in China, adapted to real-life traffic flow changes.

## 4. Parameter Selection and Analysis

This section presents the selection and analysis of various parameters for the variable approach lane signal timing optimization model and the average vehicle delay difference model. Different optimization parameters are selected through the analysis and adjusted with a certain degree of improvement to optimize the timing scheme and the functional shift scheme.

### 4.1. Signal Optimization Parameter Selection

The operational benefits evaluation parameters of an intersection usually include vehicle delay, capacity, number of stops, and queue length. As a highly relevant indicator of traffic operation benefits, vehicle delay is the most widely used indicator in traffic signal control. Meanwhile, the queue length of intersection vehicles is also one of the critical indicators for intersection operation benefit evaluation. In previous studies, scholars have conducted much research on the selection and optimization of parameters. For example, queue length, traffic delay, and carbon emission at intersections are used as traffic efficiency evaluation indicators of variable lanes to analyze the actual benefits of setting up variable lanes to mitigate intersection time and space resources [36], or to establish intersection capacity, delay optimization models [37], and vehicle emission models, etc. The traffic delay is usually calculated using the Webster or HCM models. However, the Webster model does not apply to intersections at various saturation levels, and the HCM model is too complex to be calculated easily.

Therefore, this paper optimizes the signal control scheme based on setting variable lanes to achieve collaborative control of both and thus maximize the intersection traffic benefits. A multi-objective optimization model of intersection variable-guideway signal timing with the objectives of minimizing the average vehicle delay, minimizing the queue length, and maximizing the capacity is established. In this regard, the average vehicle delay equation is optimized and improved to make the model applicable to various situations. In this paper, the model is evaluated using the queue length instead of the stopping rate indicator in most studies, which gives a more intuitive representation of the optimization effect of the model.

#### 4.1.1. Variable Approach Lane Intersection Delays

Obtaining minimum vehicle delays is the main objective of traffic signal control. In the field of traffic, a common formula for calculating vehicle delays is the Webster model as Equation (1):

(1) $$d_{i}=\frac{C{\left(1-\lambda_{i}\right)}^{2}}{2\left(1-y_{i}\right)}+\frac{y_{i}^{2}}{2q_{i}\lambda_{i}\left(\lambda_{i}-y_{i}\right)}$$

where C is the cycle length (unit s). $\lambda_{i}$ is the green ratio of phase i. $y_{i}$ is the flow ratio of phase i. $q_{i}$ is the flow of phase i (unit pcu/h). The optimum period equation is as follows:

(2) $$C_{0}=\frac{1.5L+5}{1-Y}$$

where $C_{0}$ is the optimum cycle length (unit s). *L* is total loss time (unit s). *Y* is the total flow ratio of the critical lane group.

The Webster model has a single indicator for analysis, which is not applicable in complex traffic situations. Therefore, some researchers have further optimized the model. Axelico of Australia has added a ‘stopping compensation factor’ to the Webster model. In addition, he relates this coefficient K to vehicle delays to evaluate the optimality of the intersection signal cycle and obtains the following equation for the optimal cycle:

(3) $$C_{0}=\frac{(1.4+K)L+6}{1-Y}$$

As can be seen from the formula, when *K* = 0, the formula is very close to the Webster formula. Because only the dominant traffic flows are favored in the ARRB model, the actual signal period is generally taken to be a little smaller than the optimum period obtained from the ARRB model. This helps to reduce delays in pedestrian crossings and delays in non-primary traffic.

The Webster model is a good measure of delay at low saturation, especially intersection saturation of less than 0.85. The ARRB model measures delays well at low saturations and significantly overestimates delays at high saturations.

In a study by Yang Jin-dong et al. [38], they proposed a new method for signal timing at urban road intersections. Compared with the previous method, the new method is more adapted to the traffic characteristics of intersections and more in line with the characteristics of real-time signal changes. The effectiveness of the formula is illustrated by the survey data of 12 intersections in Shanghai, and there are also relevant examples to illustrate the effectiveness of the formula for delay calculation compared to the Webster formula. Based on the optimization model mentioned in this article, this paper improves on Equation (1) by adding the effect of saturation on delay by redefining the average intersection vehicle delay as follows:

(4) $$d_{i}=\frac{c{\left(1-\lambda_{i}\right)}^{2}}{2\left(1-y_{i}\right)}+\frac{1-\lambda_{i}}{2q_{i}}+\frac{q_{i}}{2s_{i}\lambda_{i}\left(s_{i}\lambda_{i}-q_{i}\right)}$$

where $d_{i}$ is the delay for phase i (unit s) and $s_{i}$ is the saturation flow rate for phase i (unit pcu/h). C is the cycle length (unit s). $\lambda_{i}$ is the green ratio of the lane group i, $\lambda_{i}$ = $g_{i}$/C. $g_{i}$ is the effective green time of the lane group i (unit s). $y_{i}$ is the flow ratio of the lane group i, $y_{i}$ = $q_{i}$/$S_{i}$. $q_{i}$ is the flow of the lane group i (unit pcu/h).

In this paper, the optimization of the Webster delay formula not only takes into account the traffic constraints but also incorporates the traffic signal durations, which can work well with the later signal optimization.

#### 4.1.2. Capacity

The capacity of an intersection is the sum of the capacities of the intersection approach. Suppose an intersection contains *n* critical phases. The capacity is as follows:

(5) $$Q=\sum_{i=1}^{n}\frac{s_{i}g_{i}}{C}=\sum_{i=1}^{n}s_{i}\times \lambda_{i}$$

where Q is the capacity of the intersection (unit pcu/h). $s_{i}$ is the saturation flow of the critical phase (unit pcu/h). $\lambda_{i}$ is the green ratio of critical phase i. $g_{i}$ is the effective green time of critical phase i (unit s). C is the cycle length (unit s).

#### 4.1.3. Queue Length

Queues at intersections are caused by vehicles unable to cross the intersection within a given green time. The queue length is mainly determined by the traffic conditions at the intersection, and this paper investigates under-saturated intersections.

In a study by Yin Hongbin et al. [39], when the intersection is under-saturated, the queue length is the sum of the queue length before the traffic signal changes to red and the queue length of vehicles during the red light, as shown in Equation (6):

(6) $$\left\{\begin{array}{c} l=l^{*}+l_{R}\\ l^{*}=\frac{\exp\left[-\frac{4}{3}\times (\frac{g}{C}\times C\times s{)}^{0.5}\times \frac{1-x}{x}\right]}{2(1-x)}\\ l_{R}=q\times C\times (1-\frac{g}{C}) \end{array}\right.$$

where l denotes the queue length at the start of the green light in each cycle (unit m). $l^{*}$ is the queue length before the start of the red light (unit m). $l_{R}$ is the queue length accumulated during the red-light time (unit m). q is the arrival flow (unit pcu/h). *C* is the cycle length (unit s). *g* is the green time (unit s). *x* is the saturation degree.

### 4.2. Average Vehicle Delay Differential Solving and Analysis

Before signal control optimization, the functional shift setting of the intersection’s variable lanes is judged. Due to the uneven distribution of straight and left-turn traffic in the approach, one turn of traffic can sometimes be much larger than the other, with larger delays in the direction of heavy traffic, which can cause an increase in overall delay times at the intersection. To minimize delays across the intersection, the delay times for each lane should be clarified before the lane function attributes are changed, and the lane functions should be allocated reasonably to balance the delay.

This paper presents a mean delay difference model, which characterizes the difference between the mean delay of vehicles before and after a change in the function of the variable approach lane at the signal intersection. Typically, most scholars use the Webster, ARRB, or HCM models to calculate the average delay at intersection approaches. In a study by Yang Jin-dong et al. [38], they proposed a new method for signal timing at urban road intersections. Compared with the previous method, the new method is more adapted to the traffic characteristics of intersections and more in line with the characteristics of real-time signal changes. Based on the optimization model mentioned in this article, we developed an improved Webster model to solve the average delay of vehicles in the approach lane in order to apply it to the more complex traffic flow conditions.

Assuming that the delays for each lane group are generated by the random arrival of vehicles and that there is no queuing on the initial target approach lane, the average vehicle delay values for the lane groups are shown in Equation (4).

In addition to considering the change in average approach traffic delay before and after the change, the change in the functional attributes of the variable guideway is subject to certain constraints. We find that the following three conditions occur for intersection approach traffic flows:

(1) Both straight and left-turn traffic are not saturated, and no congestion occurs, so the variable approach lane function does not need to be changed.
(2) Both straight and left-turn traffic are oversaturated, at which point both the straight and left-turn lanes of the approach will generate queues of vehicles, and the intersection timing design should be readjusted.
(3) Oversaturation in one direction of both straight and left-turn traffic. When the detection of the intersection approach traffic in one direction is much larger than the traffic flow in the other direction, the lane function of the direction of the smaller traffic flow should be transformed into the direction of traffic overflow to balance the traffic flow in both directions and the road space resources.

The constraint on the change in the functional properties of the lane is expressed as follows:

(7) $$(X_{S}-x_{0S})(X_{L}-x_{0L})<0$$

where $X_{S}$ and $X_{L}$ indicate the saturation of the straight and left turn lane groups before the lane function attribute is changed, respectively. $x_{0s}$ and $x_{0L}$ denote the saturation of straight and left turn, respectively, where the average overflow queue is approximated to zero before the variable approach lane attribute is changed.

In summary, the variable approach lane vehicle average delay difference is modeled as follows:

(8) $$\begin{array}{c} z=\left(d_{S}-d_{S}^{\prime }\right)q_{S}+(d_{L}-d_{L}^{\prime })q_{L}\\ s.t.(X_{S}-x_{0S})(X_{L}-x_{0L})<0\\ \sum_{i}n_{i}=N \end{array}$$

where $d_{S}$ and $d_{S}^{\prime }$ indicate the average vehicle delay in the approach straight lane group before and after the lane function change (unit s), respectively. $d_{L}$ and $d_{L}^{\prime }$ indicate the average vehicle delay in the approach left turn lane group before and after the lane function change (unit s), respectively. $q_{S}$ and $q_{L}$ indicate the flow in the approach straight and left turn lane groups, respectively (unit pcu/h). $n_{i}$ denotes the number of lanes in the lane group. N denotes the total number of lanes in the target approach.

Since the initial functional attribute of the guide lane is straight, that is, the number of straight lanes $n_{1}$ = 3 and the number of left-turn lanes $n_{2}$ = 1 on the eastbound lane before the lane functional change, and the number of straight lanes $n_{3}$ = 2 and the number of left-turn lanes $n_{4}$ = 2 after the attribute change, the ideal basic saturation flow rate is 1650 pcu/(h⋅ln) for a single straight lane and 1550 pcu/(h⋅ln) for a single left-turn lane.

**Figure 2:**
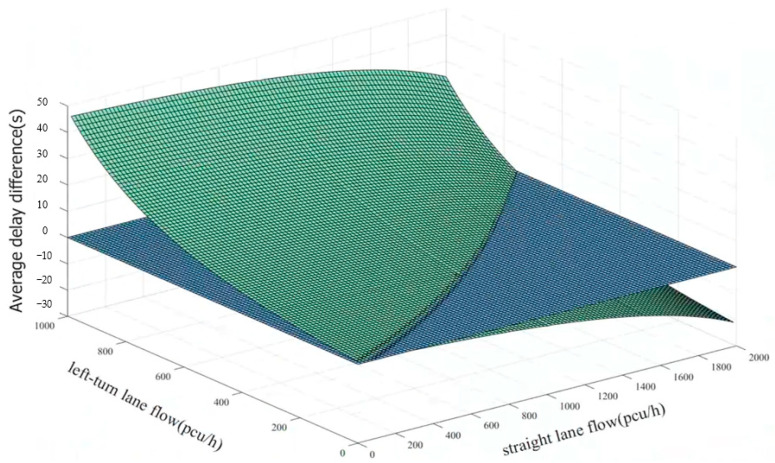
Average delay difference in the east approach before and after the lane function attribute change.

The above parameters and the current signal timing scheme are substituted into Equations (4) and (8), respectively, programmed and plotted by MATLAB (MATLAB R2021b), and solved for as shown in Figure 2.

In Figure 2, the blue plane indicates a straight–left-turn flow combination with an equal average delay before and after the lane functional attribute change. The upper area of the blue plane indicates a straight–left-turn flow combination with reduced average delay after the lane functional attribute change. The lower area indicates a straight–left-turn flow combination with an increased average delay after the lane functional attribute change.

**Figure 3:**
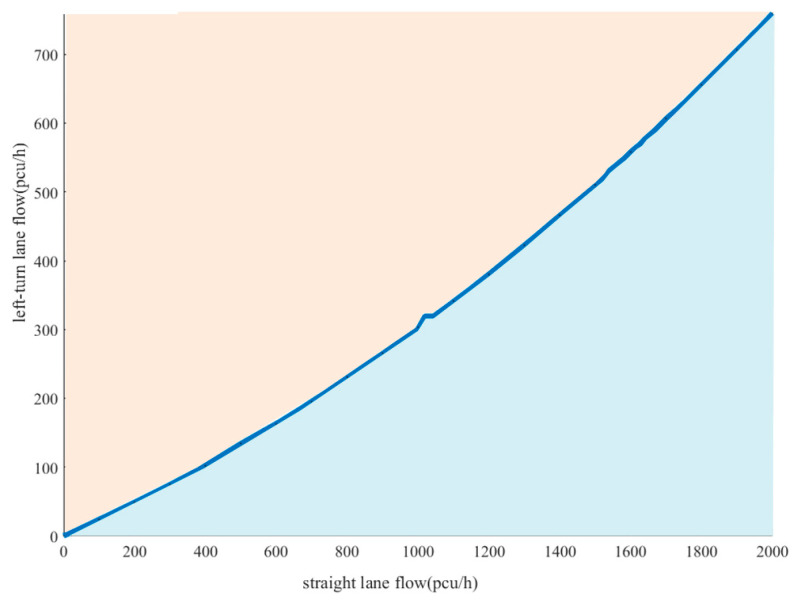
Average delay threshold curve for vehicles in the east approach.

The curve where the two planes intersect to obtain the threshold curve for the single approach variable guide lane functional attribute is shown in Figure 3. In practice, based on the real-time straight and left-turn flows and the threshold curve, it is possible to determine whether the variable guide lane functional properties need to be changed. When the combination of straight and left-turn traffic is above the threshold curve, the variable guideway function needs to be changed. When it is below the threshold curve, it remains unchanged. Representative critical values in the threshold curve were selected, and the results are shown in Table 2.

As seen from Table 2, the critical value range is between 0.20 and 0.28 for the proportion of left turn to total traffic in the approach. This indicates that when the ratio of left-turn traffic exceeds this range, the current status of the approach lane function cannot meet the demand for left-turn traffic, and the variable approach lane function needs to be changed.

## 5. Model Establishment

### 5.1. Signal Timing Optimization Model

After the parametric analysis and the results of the analysis of the function change in the variable approach lane in Section 2, based on the optimization of this spatial dimension, this paper establishes an optimization model benefit function of the time dimension with the objectives of maximum intersection variable lane capacity, minimum average delay, and minimum queue length as the following equation:

(9) $$minf\left(g\right)=\sum_{i}\left(\frac{d_{i}}{d_{0}}+\frac{l_{i}}{l_{0}}-\frac{Q_{i}}{Q_{0}}\right)$$

where $d_{i}$ is the average vehicle delay for phase i, $l_{i}$ is the queue length for phase i, and $Q_{i}$ is the capacity of phase i. $d_{0}$, $l_{0}$, and $Q_{0}$ denote the average vehicle delay, queue length, and capacity under the original signal timing scheme, respectively.

After determining the objective optimization function, the constraints on the relevant variables must be further determined, which constitutes a complete non-linear constrained optimization problem. These constraints are mainly composed of the following factors: green time, signal period, saturation degree, etc.

(1) Green time constraints

Research on traffic signal control shows that the green time of each phase greatly affects the average waiting time and evacuation speed of the intersection traffic flow. If the green time is too short, the increase in the frequency of traffic light changes will lead to an increase in the number of vehicles that start and stop at the intersection, making it difficult for vehicles to pass through the intersection quickly. If the green time for this phase is too long, the waiting time for vehicles in other phases will increase, which will also lead to an increase in vehicle delays. Therefore, the minimum green time for this signal phase should satisfy Equation (10):

(10) $$g_{i,min}\leq g_{i}\leq g_{i,max}$$

Meanwhile,

(11) $$\sum_{i=1}^{4}g_{i}+L=C$$

where the loss time *L* is set to 12 s.

(2) Cycle length constraints

Signal cycles that are too short or too long not only have a negative impact on traffic operations, but also result in vehicles not being able to cross the intersection safely and pedestrians not being able to cross the street safely [40]. Therefore, a reasonable range of signal periods should be set to limit the minimum and maximum periods in signal timing control at intersections. The limits for a complete signal cycle are given by the following equation:

(12) $$40=C_{min}\leq \sum_{i=1}^{4}g_{i}+L\leq C_{max}=120$$

(3) Saturation degree constraint

To ensure stable traffic flow at the intersection, the saturation degree is generally set not to exceed 0.9. Therefore, based on the actual traffic demand and timing situation, the maximum saturation degree of the intersection signal control is set to 0.9 [40], with the following constraint:

(13) $$x_{i}=\frac{y_{i}}{\lambda_{i}}=\frac{y_{i}}{g_{i}/C}\leq 0.9$$

where $x_{i}$ is the saturation degree of the phase i. $y_{i}$ is the flow rate of the phase i. $\lambda_{i}$ is the green ratio of the phase i.

Namely,

(14) $$g_{i}\geq \frac{q_{i}C}{0.9S_{i}}$$

Then, the signal optimization model for variable lane intersections concerning variable $g_{i}$ is as follows:

(15) $$\begin{array}{c} \min f\left(g\right)=\sum_{i}\left(\frac{d_{i}}{d_{0}}+\frac{l_{i}}{l_{0}}-\frac{Q_{i}}{Q_{0}}\right)\\ s.t.\;g_{i,min}\leq g_{i}\leq g_{i,max}\\ C_{min}\leq \sum_{i=1}^{4}g_{i}+L\leq C_{max}\\ g_{i}\geq \frac{q_{i}C}{0.9S_{i}} \end{array}$$

### 5.2. Model Optimization Results

The intersection in Figure 1 is selected for analysis. Because this intersection has an obvious vehicle flow imbalance, variable lanes are needed for optimization. The peak hour flow for each direction at the intersection is shown in Table 3. Traffic volumes are based on the open dataset for the Yellow Branch junction in the Hefei Demonstration Area [41].

Based on the peak hour flow for each direction at the intersection, it can be seen that the straight and left turn flow on the east approach are 1010 pcu/h and 430 pcu/h, respectively. Combined with the average delay threshold curve for the east approach (Figure 3), the flow combination (1010, 430) lies above the threshold curve and meets the threshold condition for lane function shift.

If a straight lane is changed to a left turn lane, the approach lane function is set as follows: 2 left-turn lanes and 2 straight lanes. In this paper, two schemes are used for signal timing design, respectively. Table 4 shows the phase and flow data for schemes I and II. The ideal basic saturation flow rate is 1650 pcu/(h⋅ln) for a single straight lane and 1550 pcu/(h⋅ln) for a single left-turn lane based on road capacity analysis [42].

Scheme I: the optimal timing method in this paper.

Based on the flow ratio and phase, the key flow ratios for each phase are calculated, and the timing scheme is adjusted using the optimized signal control method in Equation (15). The model is solved using the *fmincon* function with a non-linear multivariate minimum, and the signal timing scheme is obtained as follows:

C = 92 s, $g_{1}$ = 31 s, $g_{2}$ = 14 s, $g_{3}$ = 21 s, $g_{4}$ = 14 s

Scheme II: the Webster method.

Keeping the initial signal cycle length, the critical flow ratios for each phase are calculated based on the flow direction of each approach, and the signal timing scheme is obtained as follows:

C = 106 s, $g_{1}$ = 36 s, $g_{2}$ = 19 s, $g_{3}$ = 24 s, $g_{4}$ = 15 s

## 6. Simulation Validation

**Figure 4:**
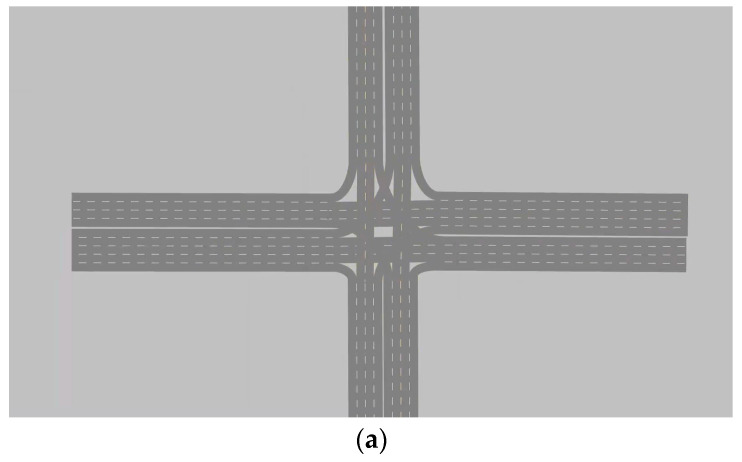

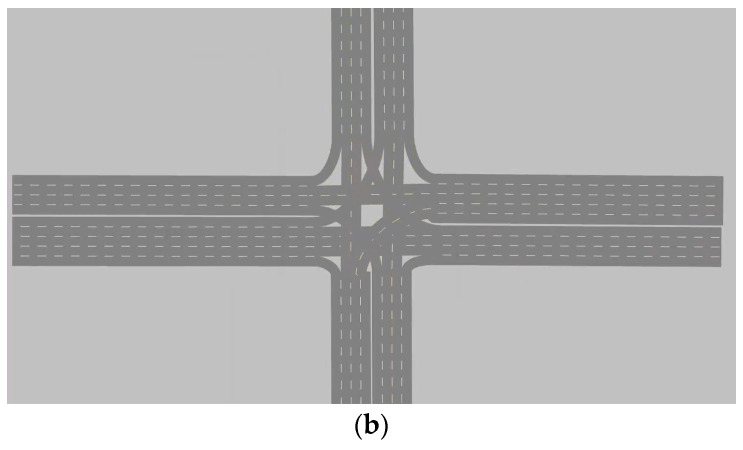
Layout of the intersection. (**a**) Variable guided lanes without lane change function. (**b**) Variable guided lanes with lane change function.

In VISSIM, the optimal timing scheme under the change in variable approach lane function, the Webster method optimal timing scheme, and the original lane function and timing scheme (noted as the original scheme) are simulated. The simulation video is shown in the Supplementary Materials section. The layout of the intersection and the different functional shifts of the variable lanes are shown in Figure 4. The layout of the intersections is based on the Huangke intersections in Figure 1, and the associated traffic volumes are entered based on actual data. Regarding the relevant parameter settings in the simulation, the parameters used are the generic default values of VISSIM since the driver’s behavior is not involved. Traffic volumes are entered according to those in Table 4, and the signal scheme is adjusted according to the timing of the two schemes in Section 4. The simulation is performed for 3600 s 10 times with a random seed of 42.

These schemes are compared using evaluation indicators such as travel time, speed, average vehicle delay, queue length, queue delay, and total pass time. Table 5 shows the comparison of traffic vehicle numbers and travel time for the three schemes.

From Table 5, it can be found that the optimized scheme in this paper has the highest total number of traffic vehicles and the lowest total travel time, which is 18.7% less than the travel time of the original scheme and 9.9% less than the travel time of the Webster scheme. This indicates that the optimized scheme in this paper can improve throughput efficiency effectively. Especially on the east–west entrance road, the volume of traffic using our scheme is highest but has the shortest passage time.

**Figure 5:**
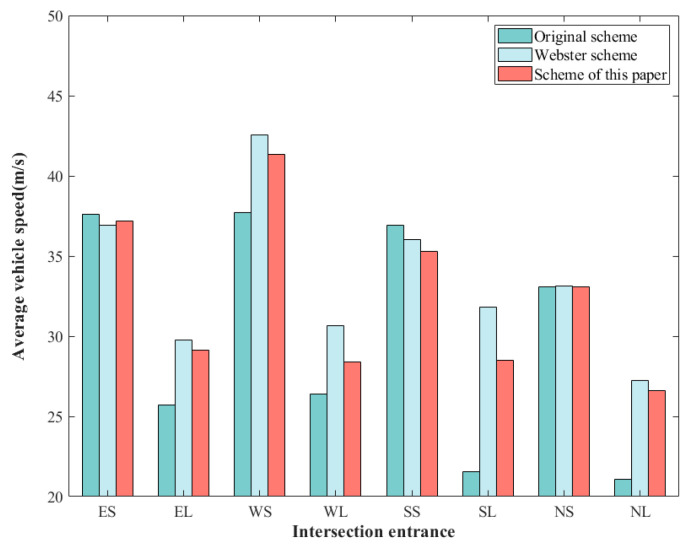
Comparison of average vehicle speed.

A comparison of the average vehicle speeds for each scheme is shown in Figure 5. In Figure 4, the east approach straight is noted as ES, the east approach left turn is noted as EL, and the rest is similar.

As seen in Figure 5, the Webster scheme has the largest average vehicle speed, and the optimized scheme in this paper has a slightly smaller average vehicle speed than the Webster scheme. The average speed of vehicles in the Webster scheme is relatively larger because the detector locations set are located in the roadway before the intersection, and the average speed calculation does not take queuing delays into account. However, if the overall signal situation is taken into account, the delay metrics in Table 6 are more indicative of the reality of congestion.

The comparison results of the average delay, static average delay, total queue length, and maximum queue length for the three schemes are shown in Table 6.

From Table 6, it can be seen that both the delay and queue length of the optimized scheme in this paper have improved to a greater extent than the Webster solution and the original solution. In particular, the average delay of this scheme is 24.3% better than the original scheme, and the queue length is 15.6% better than the Webster scheme.

The level of service of a signal intersection can effectively reflect the satisfaction of signal intersection users with the services provided by the intersection, so this paper adopts the level of service to evaluate the optimization effect of the three schemes comprehensively. The service levels for each approach direction for the original scheme, the Webster scheme, and the optimized scheme in this paper are shown in Table 7.

As seen from Table 7, the optimized scheme in this paper has improved the level of service in the ES, EL, NS, and NL approach lanes compared with the other two schemes. In contrast, the Webster scheme has the same level of service as the original scheme. In addition, the level of service is consistent with the other two scenarios in the SS and SL, which may be because this paper mainly shifts the function of the variable approach lane in the east approach and the south approach has less flow; therefore, there is a minor increase in the level of service.

## 7. Conclusions

To solve the common intersection congestion and unbalanced traffic distribution phenomenon of urban roads, this paper constructs a VAL average delay differential model and a signal timing optimization model based on the modified Webster average delay model from the two dimensions of time and space, from the perspective of VAL function change and intersection signal timing. Firstly, this paper discusses the left-turn flow threshold for VAL function switching based on actual traffic flow. The results can serve intersections in real life, effectively improve traffic efficiency, and provide references for future research. The threshold conditions for the change in VAL function attributes can be obtained by solving the model. When the proportion of left-turn traffic in the total flow on the single approach lane exceeds (0.20, 0.28), the current lane allocation cannot meet the turning needs of left-turn traffic, so the change in lane function can be considered. Based on the VAL setting, this paper further optimizes the signal control scheme. A multi-objective optimization model for the signal timing of VALs at intersections with the objectives of minimum average vehicle delay and queue length is established. The delay formula used in this paper is the direct Webster method. In this paper, we adopt an improved delay method that is more effective than the Webster method after empirical and data validation and combine the VAL function transformation to make the whole study more complete and produce greater traffic benefits. Finally, the comparison results of the optimized model in this paper with the original scheme and the scheme based on simple Webster signal timing are obtained through VISSIM experimental simulation. The results show that, compared with the original and Webster schemes, the average delay of the cooperative optimization scheme in this paper is 24.3% better than the original scheme, and the queue length is 15.6% better than the Webster scheme. The cooperative optimization method has the lowest total delay, smallest queue length, shortest travel time, largest capacity, and the best level of service, proving the feasibility and efficiency of the VAL configuration and the optimized signal timing scheme.

The methodology in this paper is verified to be effective; however, delays are still an area of inquiry worth pursuing, and more accurate public modeling of delays needs to be further developed in future research. Meanwhile, future considerations of adding economic and environmental factors to the optimization model can make the method of this paper more practical.

## Figures and Tables

**Table 1:** Intersection signal timing scheme.

Phase No.	Phase Status	Green Time [s]	Amber Time [s]	Total Cycle Length [s]
1	Straight East–West	33	3	106
2	Turn Left East–West	21	3	
3	Straight North–South	24	3	
4	Turn Left North–South	16	3	

**Table 2:** Critical flow for the change in function of the variable guideway in the east approach.

Lane Direction	Critical Flow (Ratio) [pcu/h]		Left Turn Flow Ratio
	Straight	Turn Left	
	300	74.8	0.20
	400	101.9	0.20
	500	134.4	0.21
	600	162.5	0.21
	700	195.0	0.22
	800	230.8	0.22
	900	265.5	0.23
	1000	303.4	0.23
	1100	341.3	0.24
	1200	380.3	0.24
	1300	422.6	0.25
	1400	465.9	0.25
	1500	510.4	0.25
	1600	558.1	0.26
	1700	606.8	0.26
	1800	655.6	0.27
	1900	706.5	0.27
	2000	759.6	0.28

**Table 3:** Flow of peak hours in each direction.

The Direction of Approach	Lane Function	Lane Number	Flow [pcu/h]
East	Straight	2	1010
	Turn Left	2	430
West	Straight	3	1000
	Turn Left	1	245
South	Straight	2	680
	Turn Left	1	205
North	Straight	2	560
	Turn Left	1	190

**Table 4:** Phase and flow of scheme I and scheme II.

Phase	Approach	Lane Function	Flow [pcu/h]	Saturation Flow [pcu/h/lane]	Number of Lanes	Flow Ratio	Critical Flow Ratio	Total Flow Ratio
1	East	Straight	1010	1650	2	0.31	0.31	0.81
	West	Straight	1000		3	0.20		
2	East	Left	430	1550	2	0.14	0.16	
	West	Left	245		1	0.16		
3	South	Straight	680	1650	2	0.21	0.21	
	North	Straight	560		2	0.17		
4	South	Left	205	1550	1	0.13	0.13	
	North	Left	190		1	0.12		

**Table 5:** Comparison of traffic vehicle numbers and travel time for the three schemes.

Approach Direction/Function	Original Scheme		Webster Scheme		The Scheme of This Paper	
	Number of Traffic Vehicles/pcu	Travel Time/s	Number of Traffic Vehicles/pcu	Travel Time/s	Number of Traffic Vehicles/pcu	Travel Time/s
East/ Straight	160	39.34	155	37.61	160	29.04
East/ Turn Left	35	49.94	35	50.69	34	38.4
West/ Straight	176	42.26	174	31.58	179	34.86
West/ Turn Left	64	62.39	74	44.96	70	42.99
South/ Straight	99	29.48	96	30.21	103	30.46
South/ Turn Left	31	40.96	30	39.19	31	39.49
North/ Straight	74	37.53	73	37.8	77	29.46
North/ Turn Left	19	50.55	19	46.29	19	41.96
Total	658	352.45	656	318.33	673	286.66

**Table 6:** Comparison of delay and queuing length of three schemes.

	Original Scheme	Webster Scheme	The Scheme of This Paper
Average Delay Per Vehicle [s/pcu]	34.2	29.9	25.9
Static Average Delay Per Vehicle [s/pcu]	25.9	25.2	21.1
Total Queue Length [m]	121.7	124.6	105.2
Maximum Queue Length [m]	110.7	112.7	99.4

**Table 7:** Comparison of service levels.

Approach Lanes	Original Scheme	Webster Scheme	The Scheme of This Paper
ES	C	C	B
EL	D	D	C
WS	C	B	C
WL	D	C	C
SS	C	C	C
SL	C	C	C
NS	C	C	B
NL	D	D	C

## Data Availability

All the data included in this study are available upon request by contact with the corresponding author.

## Supplementary Materials

The simulation video of three scheme is displayed in website CSDN link: https://live.csdn.net/v/414177; https://live.csdn.net/v/414178; https://live.csdn.net/v/414179 (accessed on 1 August 2024).

## References

[1] Harvey A., Bullock D. Implementation of a distributed control system for dynamic lane assignment. Proceedings of the 28th Southeastern Symposium on System Theory.

[2] Hoose H.J. (2005). Planning Effective Reversible Lane Control. TIE J..

[3] Wolshon B., Lambert L. (2006). Reversible Lane systems: Synthesis of practice. J. Transp. Eng..

[4] Zhao F., Fu L., Pan X., Zhong M., Kwon T.J. (2022). An interactive traffic signal optimization approach with dynamic variable guidance lane control. J. Adv. Transp..

[5] Gong X., Kang S. (2006). Study and application of traffic direction changing algorithm for urban tide traffic situation. J. Transp. Syst. Eng. Inf. Technol..

[6] Assi K.J., Nedal T.R. (2018). Proposed quick method for applying dynamic lane assignment at signalized intersections. IATSS Res..

[7] Pérez-Méndez D., Gershenson C., Lárraga M.E., Mateos J.L. (2021). Modeling adaptive reversible lanes: A cellular automata approach. PLoS ONE.

[8] Jiao F.T. (2018). A Dynamic Control Method for Reverse Variable Lane Based on Multi-Source Data. Master’s Thesis.

[9] Afandizadeh S., Jahangiri A., Kalantari N. (2012). Identifying the optimal configuration of one-way and two-way streets for contraflow operation during an emergency evacuation. Nat. Hazards.

[10] Nassiri H., Edrissi A., Alibabai H. (2010). Estimation of the logit model for the online contraflow problem. Transport.

[11] Xie C., Mark A.T. (2011). Lane-based evacuation network optimization: An integrated lagrangian relaxation and tabu search approach. Transp. Res. Part C Emerg. Technol..

[12] Hausknecht M., Au T.C., Stone P., Fajardo D., Waller T. Dynamic Lane reversal in traffic management. Proceedings of the 2011 14th International IEEE Conference on Intelligent Transportation Systems (ITSC).

[13] Wang X., Wang Y.L., Zhang M.C. Empirical study on Reversible Lane in Beijing. Proceedings of the International Conference on Computer Information Systems and Industrial Applications.

[14] Liu C., Yang H., Ke R., Wang Y. (2022). Toward a Dynamic Reversible Lane Management Strategy by Empowering Learning-Based Predictive Assignment Scheme. IEEE Trans. Intell. Transp. Syst..

[15] Malekzadeh M., Papamichail I., Papageorgiou M., Bogenberger K. (2021). Optimal internal boundary control of lane-free automated vehicle traffic. Transp. Res. Part C Emerg. Technol..

[16] Jin X., Yu X., Hu Y., Wang Y., Papageorgiou M., Papamichail I., Malekzadeh M., Guo J. Integrated control of internal boundary and ramp inflows for lane-free traffic of automated vehicles on freeways. Proceedings of the 2022 IEEE 25th International Conference on Intelligent Transportation Systems (ITSC).

[17] Malekzadeh M., Troullinos D., Papamichail I., Papageorgiou M., Bogenberger K. (2024). Internal boundary control in lane-free automated vehicle traffic: Comparison of approaches via microscopic simulation. Transp. Res. Part C Emerg. Technol..

[18] Xie X., Dong L., Gu H., Li H., Zhang L. (2023). A collaborative method on reversible lane clearance and signal coordination control in associated intersection. J. Adv. Transp..

[19] Zhou H., Ding J., Qin X. (2016). Optimization of variable approach lane use at isolated signalized intersections. Transp. Res. Rec. J. Transp. Res. Board.

[20] Liu W., Xie Z., Chen K. (2018). Optimization of Reversing Variable Lane Signal Timing Design Based on NSGA-Ⅱ Algorithm. J. Chongqing Jiaotong Univ. (Nat. Sci.).

[21] He S.L., Wang W., Zhang J., Yang J. (2013). An improved optimization method for isolated signalized intersection based on the temporal and Spatial Resources Integration. Procedia—Soc. Behav. Sci..

[22] Cui S., Xue Y., Gao K., Wang K., Yu B., Qu X. (2024). Delay-throughput tradeoffs for signalized networks with finite queue capacity. Transp. Res. Part B Methodol..

[23] Lu T., Yang Z., Ma D., Jin S. (2018). Bi-level Programming Model for Dynamic Reversible Lane Assignment. IEEE Access.

[24] Hong W., Yang Z., Sun X., Wang J., Jiao P. (2022). Temporary Reversible Lane Design Based on Bi-Level Programming Model during the Winter Olympic Games. Sustainability.

[25] Wong C.K., Heydecker B.G. (2011). Optimal allocation of turns to lanes at an isolated signal-controlled junction. Transp. Res. Part B Methodol..

[26] Zhuo J., Zhu F. (2023). Evaluation of platooning configurations for connected and automated vehicles at an isolated roundabout in a mixed traffic environment. J. Intell. Connect. Veh..

[27] Xuan Y., Daganzo C.F., Cassidy M.J. (2011). Increasing the capacity of signalized intersections with separate left turn phases. Transp. Res. Part B Methodol..

[28] Fu L.J., Guo H.F., Dong H.Z. (2011). Variable Lane adaptive control method based on dynamic traffic flow. Sci. Technol. Bull..

[29] Zhao J., Ma W., Zhang H.M., Yang X. (2013). Increasing the capacity of signalized intersections with dynamic use of exit lanes for left-turn traffic. Transp. Res. Rec. J. Transp. Res. Board.

[30] Yuan Q., Shi H., Xuan A.T., Gao M., Xu Q., Wang J. (2023). Enhanced target tracking algorithm for autonomous driving based on visible and infrared image fusion. J. Intell. Connect. Veh..

[31] Xue Y., Wang C., Ding C., Yu B., Cui S. (2024). Observer-based event-triggered adaptive platooning control for autonomous vehicles with motion uncertainties. Transp. Res. Part C Emerg. Technol..

[32] Tian Y.Q., Shang Z.H. (2013). Research on the variable lane setting of the exit road at urban road intersections. Urban Transp..

[33] Zhao J., Zhao X.Z. (2016). The optimal lane function and signal conversion method of variable lanes at intersections. J. Univ. Shanghai Sci. Technol..

[34] Qu D.Y., Cao J.Y., Wang P., Li J., Xu X. (2017). Green wave control method for coordinated optimization of tidal lanes and changing lanes. J. Jinan Univ..

[35] Chen Y.Y., Han W. Multi-objective signal timing optimization method for reverse variable lane intersections. Proceedings of the CICTP 2022.

[36] He J., Zhu Y., Zhang J., Ma X. Reversible Lane control system with low emission load based on VISSIM simulator. Proceedings of the 2021 IEEE 2nd International Conference on Big Data, Artificial Intelligence and Internet of Things Engineering (ICBAIE).

[37] Ren Q.L., Tan L.P. (2020). Signal Timing Optimization Method for Reverse Variable Lane Intersection. J. Transp. Syst. Eng. Inf. Technol..

[38] Yang J.D., Yang D.Y. (2001). Optimization model of signal cycle time of urban signal control intersection. J. Tongji Univ. Nat. Sci. Ed..

[39] Yin H.B., Xu J.-m. (2000). Road Traffic Control Technology.

[40] Wu B., Li Y. (2015). Traffic Management and Control.

[41] Sha Z., OpenITS Org (2021). OpenData 6.1-Introduction of HuangKe Intersection Data in Hefei Demonstration Area. http://www.openits.cn/openData2/710.jhtml.

[42] Chen K.M., Yan B.J. (2003). Road Capacity Analysis.

